# Supplementary figures and images for: Deep learning classification of lipid droplets in quantitative phase images
Source: PLoS One. 2021 Apr 5;16(4):e0249196. doi: 10.1371/journal.pone.0249196 (PMC8021159; doi:10.1371/journal.pone.0249196)

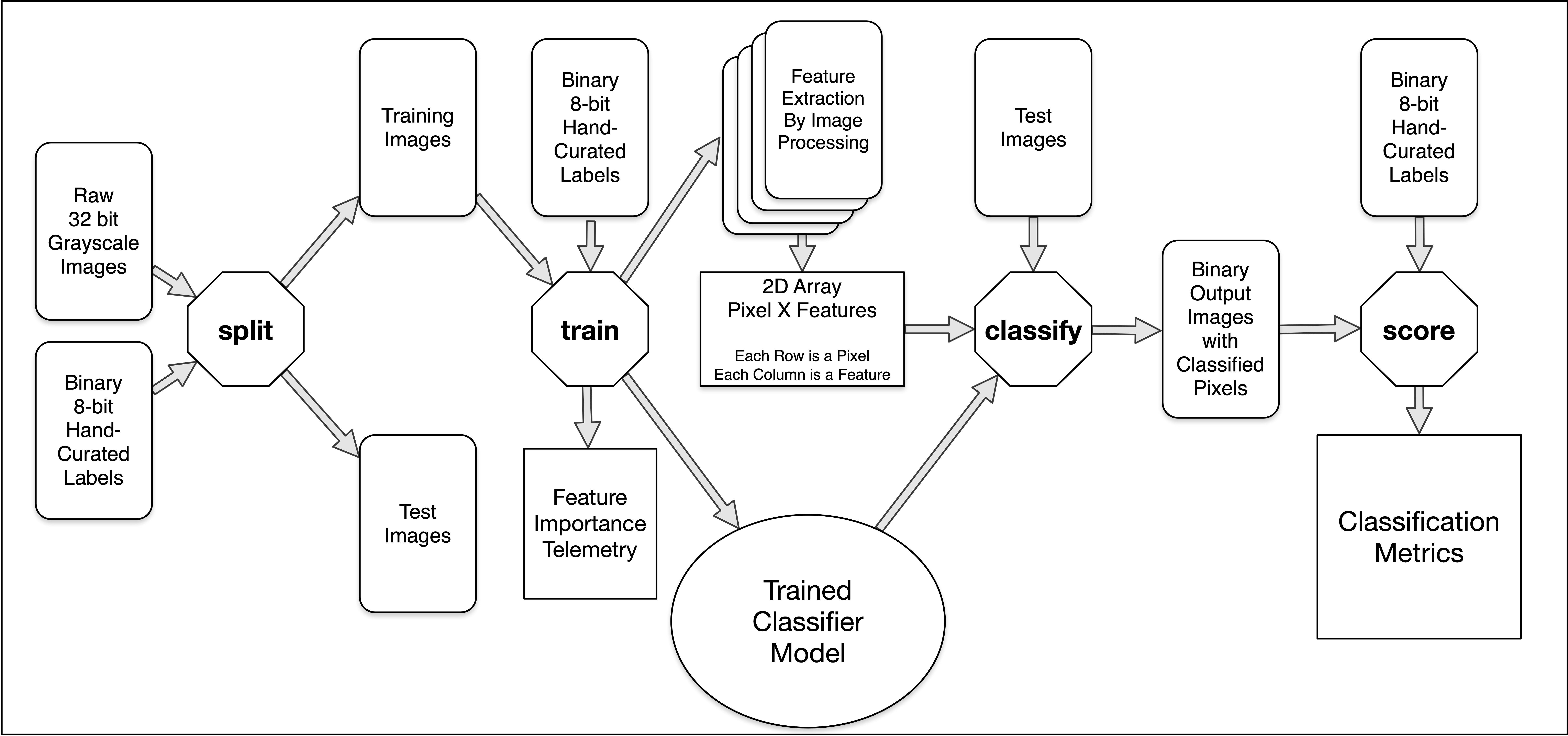

Supplement: S1 Fig — (PNG) [file pone.0249196.s001.png]
